# Preanaesthetic assessment and management in the context of the district hospital

**DOI:** 10.4102/safp.v63i1.5357

**Published:** 2021-09-07

**Authors:** Olufemi B. Omole, Michelle Torlutter, Agetta J. Akii

**Affiliations:** 1 Department of Family Medicine, Faculty of Health Sciences, University of the Witwatersrand, Johannesburg, South Africa

**Keywords:** Preanaesthetic, assessment, district hospital, review, management

## Abstract

Preanaesthetic assessment and management allow for the systematic identification of perioperative risks and the implementation of interventions to mitigate them, such that the patient’s physiological state is optimised for surgery or other procedures. This is a crucial activity for good perioperative outcomes, as patients not assessed are at a higher risk of unanticipated adverse perioperative events and are more likely to receive suboptimal management. The district hospitals in South Africa perform minor and moderately complex surgical procedures that require anaesthesia, administered mostly to healthy patients and those with stable diseases without functional limitations. A significant proportion of anaesthesia-related deaths reported in the district hospitals can be linked to poor risk assessment and management. In this article, we highlight the key clinical imperatives for optimal preanaesthetic assessment and management from the district hospital perspective.

## Introduction

Preanaesthetic assessment is the process of clinical evaluation that precedes the administration of anaesthetics for surgical and non-surgical procedures.^1^ Apart from providing an opportunity to build good doctor–patient rapport, it allows for the systematic identification of the patient at risk of adverse perioperative events and the implementation of targeted interventions to optimise favourable perioperative outcomes.^2^ Achieving the above is a clinical and policy imperative in South Africa district hospitals, where according to the 2017 Saving Mothers report, 65.5% of all anaesthetic deaths were reported.^3^ Furthermore, preanaesthetic assessment reduces patient anxiety about perioperative care, the rate of surgery cancellation or postponement and the length of hospital stay.^4^ In line with international standards, the Society of Anaesthesiologists of South Africa (SASA) affirms that every patient presenting for anaesthesia should have a preanaesthetic medical assessment;^5,6^ however, the extent to which this is true in South Africa is not known. It should be noted that patients who are not assessed preoperatively are at risk of suboptimal anaesthetic management.

The following are the objectives of the preanaesthetic assessment:^7,8^

- to build a good rapport between the anaesthetist and the patient, which enables shared understanding of the anaesthetic plans and perioperative care. This reduces patient’s anxiety
- to identify and assess risks associated with the administration of anaesthesia
- to eliminate or ameliorate risks by optimising patient’s health status. This may involve referral to other specialists
- to obtain consent for anaesthesia
- to order tests, premedication and other indicated interventions.

This continued professional development article considered the norms and standards for the South African^9^ district hospital surgical services package, and the recommendations of the SASA for the General practitioner Anaesthesiologist, including Diplomates. To this end, patients to be anaesthetised in South African district hospitals should mainly be American Society of Anaesthesiology (ASA)-1 and -2 (E) risk categories. Some ASA-3 risk category patients may be anaesthetised by an experienced Diplomate in Anaesthesia depending on the urgency and nature of the surgery, the availability or unavailability of supporting specialist anaesthetist and adequacy of clinical support services.^6^

## Responsibility and timing for the preanaesthetic assessment

The responsibility for preanaesthetic assessment lies with the anaesthetist. Whilst it should be preferably performed by the anaesthetist who is delivering the anaesthetics,^6^ it may be conducted by a peer anaesthetist in settings where there are dedicated preanaesthetic assessment clinics.^10^ Although there is no conclusive evidence that one approach is superior to the other in terms of outcomes,^5^ the former allows for building of rapport between the anaesthetist and the patient and/or next of kin, ensures continuity of care and minimises opportunities for missed findings.^11^

The use of a standardised health questionnaire and forms may assist in reducing variations in the quality of documentation of information during the assessment.^12^ Self-administered, electronic questionnaires have also been found to be acceptable to patients.^13^

The timing of assessment is influenced by complexity of the anticipated procedure, whether the procedure is an emergency, and the patient’s health status. In order to avoid unnecessary postponement and cancellation in elective procedures, patients who are at extremes of ages, have a poor health status and who are undergoing major and complicated procedures should be assessed as early as possible (preferably a few days before) to allow time for optimisation. For reasons of limited knowledge, skills and supportive equipment, patients at extremes of ages and those for complicated surgical procedures are not appropriate candidates for anaesthesia at the district hospital. In emergencies, the time of assessment and intervention is guided by the nature of the pathology and the urgency of surgical intervention.

## Details of the preanaesthetic assessment

The evaluation is based on multiple information sources that may include medical interview(s) with the patient and/or next of kin, physical examination, medical record appraisals, ordering and reviewing of test results, and as necessary, consultation with other healthcare professionals involved in the perioperative care.^1,7,10,14^ At the end, patients should be assigned an ASA risk category, and a management plan should be developed.

### Medical history

- *Past and present medical histories* need to be fully explored. Several perioperative complications and adverse outcomes are linked to co-morbidities such as diabetes mellitus, chronic pulmonary diseases, hypertension, heart failure, past myocardial infarction, and cerebrovascular and peripheral vascular diseases.^7,14^ Other issues to pay attention to include bleeding disorders, thyroid dysfunctions, smoking history, alcohol-use disorders and high body mass index. Previous hospitalisations, exercise intolerance, dyspnoea at rest and orthopnoea may indicate poor cardiovascular reserve. Table 1 shows the peri-anaesthetic implications of some uncontrolled medical conditions. Whilst controlling medical conditions may require postponement or delay in elective procedures, it may not be feasible to do so in emergencies. Uncontrolled medical conditions with functional limitations constitute at least ASA-3/3E risk category, and anaesthesia in such patients is best delivered in secondary or tertiary levels of care, where there are specialists and more advanced monitoring and management resources.

**TABLE 1:** Common medical conditions and associated risks in the perioperative period.

Condition	Peri-anaesthetic and clinical implications
Uncontrolled hypertension	Exaggerated cardiovascular response resulting in: Raised BP during laryngoscopy and intubation, swinging blood pressure, with an increased risk of myocardial infarction and cerebrovascular ischemia and haemorrhage Electrolyte derangement from use of diuretics.
Recent myocardial infarction in the last 3 months	Increased risk of reinfarction. Recent history or cardiovascular instability should trigger referral to a secondary or tertiary level of care.
Heart failure	Problems with fluid control and cardiac contractility and output. Is a significant predictor of adverse events in the perioperative period. Anaesthetics for these patients, especially those with NYHA class II (mild limitation of daily activities) or more severe disease, should not be managed in the district hospital because they require echocardiograph and cardiology consult preoperatively, invasive monitoring, intensive care unit (ICU) postoperatively and should be managed by specialist anaesthesiologists.
Peripheral vascular disease	Is a pointer to the presence of other cardiovascular diseases. Check for smoking, diabetes, hypertension and hypercholesterolaemia
Cardiac dysrhythmias	Increased risk of thromboembolic events, heart failure, sudden cardiac arrest and increased risk of bleeding from use of anticoagulants. May require anticoagulation (check clotting profile), rate control, invasive monitoring, ICU and advanced life support
Diabetes mellitus	Increased risk of cardiovascular and renal events, especially with history of ischaemic heart/peripheral vascular diseases.
Neuromuscular disorders	Other skeletal muscles (respiratory and cardiac) and congenital disorders may be involved. May have exaggerated response to muscle relaxants, especially non-depolarising types. May require ventilatory support postoperatively May have poor sputum clearance resulting in respiratory infections and atelectasis.

*Source*: Please see the full reference list of the article Klocke M. How to do a pre-anaesthetic assessment. In: Mash B, Blitz J, editors. South African family practice manual. 3rd ed. Pretoria: Van Schaik, 2015; p. 422–425, for more information

NYHA, New York Heart Association.

Particular attention should be paid to any history of difficulty in opening the mouth, swellings and dental problems in head and neck surgery; snoring or sleep apnoea, hypertension, obesity, nausea and vomiting in ear, nose and throat (ENT) surgery; symptoms of gastroesophageal reflux, nausea, vomiting, features suggesting bowel obstruction and anaemia in gastrointestinal surgery; and nausea, reflux and anaemia in obstetric and gynaecological surgery.

Autoimmune diseases, such as rheumatoid arthritis, ankylosing spondylitis, scleroderma and degenerative osteoarthritis in the elderly, may affect the temporomandibular joints (TMJs) and cervical and lumbar spines, thereby limiting mouth opening, neck and back movements, and making airway management and axial blocks difficult.

- Explore for a *positive family history of adverse reactions* to anaesthetics, such as malignant hyperthermia, scoline apnoea, porphyria, haemoglobinopathies and other familial diseases.
- Details of *past surgeries and anaesthesia*, anaphylaxis or drug reactions, malignant hyperthermia, scoline apnoea, postoperative nausea and vomiting (PONV), acute porphyria, emergence delirium, and so on, should be explored. In patients who have had no previous anaesthesia, family history of adverse anaesthetic events may be useful in alerting the anaesthesiologist to the possibility of risks.
- *Drug history*: Apart from allergies, obtain a list of medications the patient is on, and note the implications for the anaesthesia and surgery. Common ones to enquire about include antiplatelets, anticoagulants, antihypertensives, antiarrhythmics, other cardiac drugs and medications taken for mental health and thyroid disorders. Where appropriate, also probe for illicit drug use.
- *Pregnancy*: The possibility of pregnancy should be sensitively explored in all women of reproductive age and a test ordered for confirmation, if necessary.
- History of *last meal intake*: This is very important to prevent bronchial aspiration. For elective procedures in adults, the fasting period is dependent on the type of last meal, age group, pathology and other factors. The recommended fasting duration and factors affecting gastric emptyingare shown in Table 3.^15,16,17^

### Physical examination

Physical examination should augment history in identifying risks that may negatively affect intra- and post-operative outcomes.^7,14,16,18,19^ The focus of physical examination should be on the cardiorespiratory system. The need for other system examination is dictated by the type of surgery, patient’s health status and anticipated anaesthetic technique (general, regional block, sedation or monitored or balanced). At minimum, physical examination should include the following:

- measurement of *vital signs* (blood pressure, heart rate, temperature, weight and body mass index)
- examination of *airways, lungs, heart*, musculoskeletal system including any neurological deficit, when regional or peripheral nerve block is being contemplated.

#### Airway assessment

This is aimed at determining the ease of airway management during anaesthesia. The SASA Airway guideline (2014) recommends the following five pertinent questions to be answered for adequate airway management assessment:^20^

**FIGURE 1:**
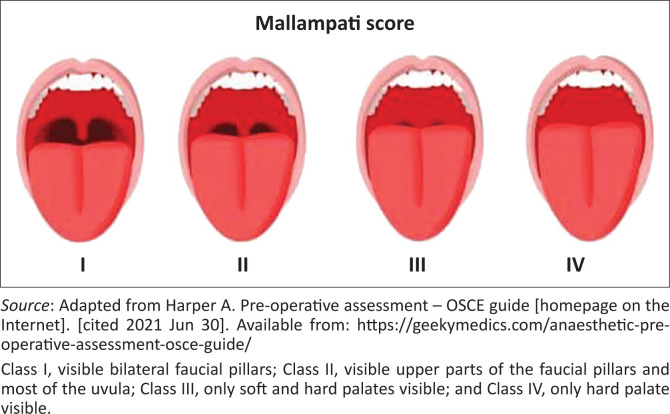
Mallampathi score.

1. *Are there any problems with consent and/or co-operation*? Uncooperative patients make preanaesthetic assessment difficult and suboptimal, and may jeopardise appropriate planning for airway management.
2. *Will laryngoscopy be difficult*? There are four areas to be explored, referred to as the 4Ds: *D*isproportions and *D*istortions in facial and airway structures, *D*ysmobility of joints in the head and neck, and abnormal *D*entition.
3. *Will mask ventilation be difficult*? Look for ‘BONES’ – *B*eard, *O*besity, *N*o teeth, the *E*lderly and Snoring patients.
4. *Will intubation be difficult*? Previous history of prolonged endotracheal intubation, voice changes, dyspnoea and reduced effort tolerance.
5. *Will airway rescue be difficult*? For difficulty with rescue with supraglottic devices, think about ‘RODS’ – *R*estricted mouth opening, *O*bstruction at the level of the larynx, *D*istortion for anatomically based devices and *S*tiff lungs. With regard to infraglottic devices, think about ‘SHORT’ – *S*urgery and *Ha*ematoma on the neck that distorts anatomy, *O*besity or abscess, past neck *R*adiations and *T*umours including goitre.

- *Mouth opening*: The inter-incisor distance should be > 3 cm or two fingers when mouth fully opened. Shorter distances, craniofacial abnormalities, protruding incisors, malocclusions, receding mandible, high-arched palate, oropharyngeal infections, angioedema of the face and mouth, loose teeth, fractures of the face involving the mandible and maxilla, and a large tongue are all associated with difficulty in intubation and airway maintenance.
  ■ *Upper lip bite test*: Movement of lower incisors forward ahead of upper incisors estimates range of temporomandibular joint during a jaw thrust. The findings of this test are classified as I to III, in increasing order of difficulty in intubation. Class I, lower incisors can bite the upper lip above the vermilion line; Class II, lower incisors can bite the upper lip below vermilion; and Class III, the lower incisors cannot bite the upper lip.
  ■ *Head and neck*: Large heads as in hydrocephalus may be cumbersome and restrict neck flexion and alignment of the airway, especially in children. Check for ranges of neck flexion, extension and lateral movements. Cervical spine fractures, previous neck surgeries, radiation to the neck and cervical fusion may affect neck movements and be associated with difficulty in airway management. Neck swellings and masses may displace the airway, and make intubation and airway management difficult.
  ■ *The thyro-mental distance* (Patil test) should be at least 7 cm, and a distance of 6 cm predicts 75% difficult laryngoscopy. Is the chin able to touch upper sternum? Check the sterno-mental distance (from the sternal notch to the tip of mandible, neck fully extended). Normal > 12.5 cm. A neck circumference greater than 27 cm may suggest poor glottic visualisation.^16,19^
  ■ Perform a *Mallampati classification* (Figure 1)^14^: Intubation becomes more difficult from Mallampati classes I–IV; however, the presence of a high Mallampati score does not necessarily preclude district hospital anaesthesia. A comprehensive assessment of the airway and availability or lack thereof, of requisite skills and equipment for advanced airway management should be performed to determine whether difficulties with mask ventilation, supraglottic device rescue and intubation are likely to be encountered. If this is the case, the patient should be transferred to a regional or tertiary level of care.
  ■ *Other considerations*: Overgrown beards, stridor, hoarse voice, orthopnoea, drooling, dysphagia, neck trauma and evidence of burns to the airway may predispose to difficulty in airway management. Other special conditions that may lead to difficulty in airway management include:
- *Pregnancy*: increased weight, facial oedema, laryngeal oedema (especially in severe pre-eclampsia and eclampsia) and large breasts.
- *Morbidly obese*: BMI > 30 kg/m^2^ is associated with increased difficulty in airway management and other perioperative risks. Although a comprehensive perioperative risk assessment should inform whether a patient should be or should not be anaesthetised at a given level of care, the lack of advanced airway management skills and equipment necessitates that adults with BMI > 40 kg/m^2^ should preferably not be anaesthetised at the district hospital.^7^
- *Elderly*: Edentulous state and arthritis (osteoarthritis, rheumatoid arthritis and ankylosing spondylitis) may affect the ability to maintain the airway. The latter may affect the cervical spines and TMJ, restricting mouth opening and neck flexion. Arthritis affecting the vertebrae may also distort the anatomy and make lumbar epidural and spinal block more difficult to perform.
- *Children*: Airway control may be challenging because of their small sizes, more cephalad and anterior vocal cords, and large tongues and heads in comparison with their bodies. Considering the highly specialised skills required, neonates and children weighing < 10 kg should not be anaesthetised in the district hospital.^7^

## Cardiovascular system

The blood pressure (BP), heart rate (HR) and pallor should be checked. Pulses should be palpated for rate, regularity and collapsing pulse. Whilst irregular pulse may indicate atrial fibrillation or ventricular ectopics, a collapsing pulse may indicate aortic incompetence. The jugular vein should be assessed for any elevated venous pressure that may suggest heart failure.

The precordium should be palpated for thrills (that may indicate clinically significant murmur of at least 4/6) and lower parasternal heave (that may indicate a hyperdynamic right ventricle), and percussed for heart size to exclude cardiac hypertrophy. Auscultate cardiac sounds to exclude clinically significant murmurs. If you suspect any pathology, perform further investigations such as electrocardiography (ECG), chest radiograph (CXR) and Echocardiograph as appropriate, including referring for specialist evaluation. Significant cardiac pathology, presence of effort intolerance, history of myocardial infarction, and signs of heart failure and cardiac arrhythmias all suggest high perioperative, cardiovascular risk and should prompt referral of such patients to a regional or tertiary centre for specialist anaesthetic management. Even in emergencies, patient should be stabilised and referral arranged.

## Respiratory system examination

Respiratory system examination includes checking for the respiratory rate, oxygen saturation rate, central and peripheral cyanosis, signs of upper airways obstruction – stridor, drooling, submandibular or oral or neck masses, and tracheal deviation.

Inspect the chest. An increased anteroposterior diameter may indicate underlying obstructive airway disease. Kyphosis or scoliosis presents with significant alteration of respiratory mechanics and gaseous exchange, and may require advanced anaesthetic management and critical care postoperatively. Such patients should not be anaesthetised in the district hospital.

Palpate and percuss the chest and auscultate lungs for good bilateral air entry in all lobes, and added breath sounds. If any problem is suspected, a chest radiograph is performed, and if necessary, the patient is referred to a physician consult.

## Musculo-skeletal and neurological system

- Cervical spine: check for ranges of movements and tenderness. In trauma cases, *do not remove rigid collar before c-spine fracture is excluded*.
- Thoracic spine: Check for kyphoscoliosis.
- Lumbar spine: Check for kyphoscoliosis, spina bifida, degenerative diseases and other deformities.
- Muscles: Check for wasting and fasciculation
- A brief neurological examination should check for muscle bulk, tone, power and reflexes, especially when a neurological problem is suspected, and before nerve blocks. Document the findings and avoid nerve and regional blocks.

## Risk assessment and categorisation

After history and examination (and when appropriate, test result reviewed), the patient should be classified into a risk category:

- *Functional status* or *exercise tolerance*: This is perhaps the single most useful risk index. It is commonly measured in metabolic equivalents (METS). One MET is the energy consumed by the body at rest (3.5 mL of oxygen consumed per kilogram body weight per minute). The capacity to climb a flight of stairs corresponds to a moderate exercise capacity and is equivalent to four METS. This is easily measured, takes less than 70 s to complete and is a sensitive cardiovascular risk index. Patients with an exercise capability of four METS or greater present with a lower risk of cardiovascular morbidity.^21^ It should be noted that some view the stair-climbing test as a test of anaerobic rather than aerobic capacity. It is therefore better tests lung function than cardiovascular function. Fatigue on a stair-climb is thus worrying; however, the ability to complete one may be falsely reassuring. For this reason, some anaesthetists prefer a 6-min walk test on the flat, which is a sub-maximal exercise test used to assess aerobic capacity and endurance.^22^
- American Society of Anaesthesiology physical status classification (Table 2)^16,19^: The ASA physical status classification was originally intended to predict operative risk and generally correlates well with the perioperative mortality rate; however, underlying disease is only one of many factors that contribute to perioperative complications, and therefore, the correlation is not perfect.^19^

**TABLE 2:** Preoperative physical status classification according to the American Society of Anaesthesiology.

Class	Definition
1	A normal healthy patient.
2	A patient with mild systemic disease and no functional limitations.
3	A patient with moderate to severe systemic disease that results in functional limitation.
4	A patient with severe systemic disease that is a constant threat to life and functionally incapacitating.
5	A moribund patient who is not expected to survive 24 h with or without surgery.
6	A brain-dead patient whose organs are being harvested.

*Source*: Allman KG, Wilson LH. Pre-operative assessment and preparation for anaesthesia. Oxford textbook of anaesthesia. New York: Oxford University Press; 2011

## Clinical investigations

**FIGURE 2:**
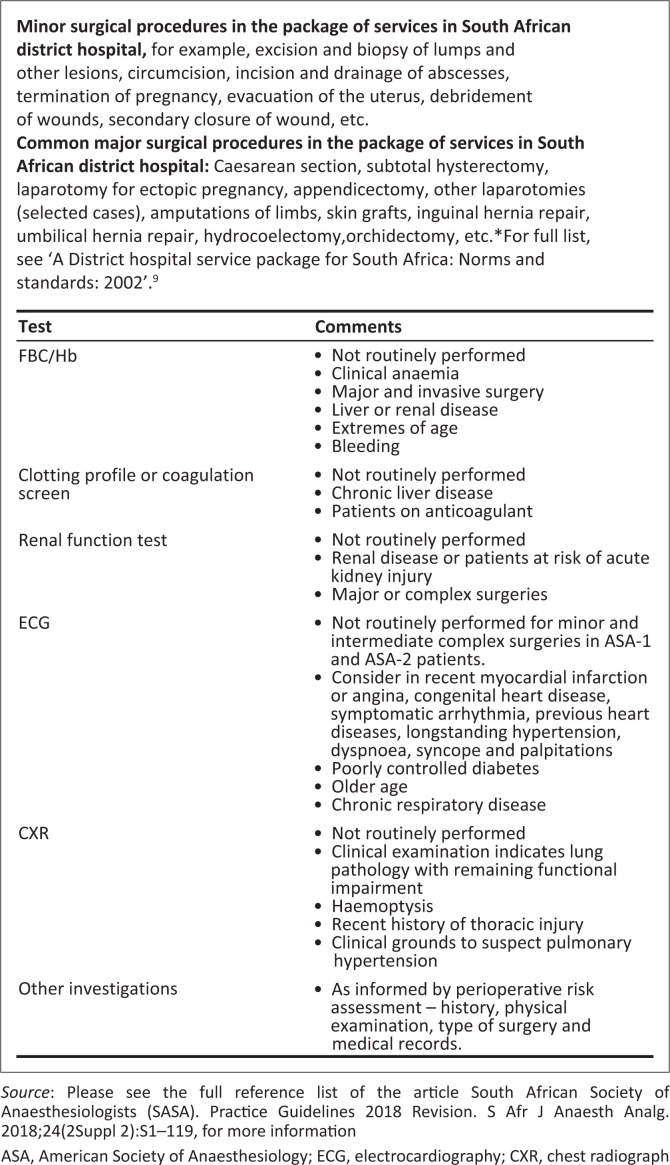
Pre-anaesthetic laboratory investigations in the district hospital.

Most surgeries performed in the district hospitals are minor or moderately complex, and up to ASA-3 risk category patients, where there is an experienced Diplomate.^23,24,25,26,27^ Routine laboratory tests are therefore not warranted, except when indicated by findings on history and examination, and the complexity of surgery. The incidence of abnormal test results is extremely low, and the need for tests should balance risk and costs against benefits. The use of point of care tests should also be considered. Figure 2 provides some guidance on investigations that can be performed in exceptional cases.

## Preanaesthetic management and premedication

The management plan will depend on the health status of the patient and the type of surgery.

- **Consent:** The findings of the risk assessment and the anaesthetic plan should be discussed with the patient for shared understanding. Enough information should be given to ensure that informed written consent can be said to have been obtained.
- **Fasting:** This is to prevent bronchial aspiration of gastric content. All patients must be fasted before anaesthesia, except in emergencies where this risk is managed along with the surgery. Guides on periods of fasting in different circumstances and the factors that may delay gastric emptying are shown in Table 3. New evidence suggests that up to 3 mL/kg clear fluids may be allowed up to 1 h before surgery in children.^15,16,17^
**Premedication**
- Although not always routinely used, premedications are still prescribed for anxious patients (Table 4)^16,17,18,19,28,29,30^. However, a good doctor–patient rapport and a clear explanation of the anticipated anaesthetic events provide more anxiolysis than drugs.^8,16^
- Intestinal prokinetic and antiemetic drugs such as metoclopramide are prescribed to facilitate gastric emptying and prevent and reduce the risk of aspiration. Proton pump inhibitors and sodium citrate are prescribed to reduce gastric acid production and increase pH, respectively.
- Prophylactic antibiotics are used preoperatively according to the South African Antibiotic Stewardship Programme guidelines. Special attention should be given to the need to prevent infective endocarditis in applicable patients with damaged or artificial heart valves, intravenous drug users, and dental, urogenital, gastrointestinal and instrumentation procedures.^31^
- *Intravenous line* ensures access for rapid administration of drugs and fluids. Dehydration is undesirable in any form of anaesthesia and should be avoided. The administration of a pre-load/ co-load of 10 mL/kg – 20 mL/kg of warm crystalloid before spinal block mitigates hypotension from the spinal block. Any fluid deficit from fasting should be estimated (4 mL/kg per hour for the first 10 kg of body weight, plus 2 mL/kg per hour for the next 10 kg of body weight plus 1 mL/kg per hour for the remaining weight in kilograms). For example, in a 40 kg young woman who has been fasted for 8 h without any fluid, the deficit is estimated as (4 × 10 × 8) + (2 × 10 × 8) + (1 × 20 × 8) = 640 mL of crystalloid. This Holliday-Segar formula may overestimate fluid requirements in the adults, and about one-half to two-third of the estimated amount is given. Any ongoing and third space loss should be added.

**TABLE 3:** Fasting guidelines in elective patients (*ASA guidelines 1999).

Ingested material	Minimum fasting duration (hours)
Meal with high fat or meat content	8 or more in adults, 6 h in children
Light meals	6 in adults
Breast milk – no additions to pumped breast milk allowed	4
Infant formula or non-human milk	6
Clear fluids (water, clear fruit juice without pulp, non-fizzy sports drinks, tea and coffee black or with a maximum 10 mL of milk, and non-carbonated drinks)	Up to 2 h in adult and 1 h in children

*Source*: Lerman J. Preoperative assessment and premedication in paediatrics. Eur J Anaesthesiol. 2013;30:645–650. https://doi.org/10.1097/EJA.0b013e328360c3e2

Note: Causes of delayed gastric emptying: metabolic causes, for example, diabetes mellitus, renal failure, sepsis; decreased gastric motility, for example, head injury and trauma; bowel obstruction; raised intra-abdominal pressure, for example, pregnancy and obesity; drugs, for example, opioids; severe trauma and pain; and gastro-oesophageal reflux, may be associated with delayed emptying of solids but not liquids.

**TABLE 4:** Drugs used for premedication.

Desired Effect	Drug	Comment
Sedatives (anxiolytic/amnestic)	Midazolam, Temazepam, Oxazepam, Diazepam	Benzodiazepines have a large interindividual variation
Gastric pH increasing drugs	Antacids – sodium citrate Proton pump inhibitors – omeprazole H2 antagonists – ranitidine	Neutralise acid in the stomach, reducing the risk of damage from aspiration Mainly use antacids given 10 min preoperatively in high-risk cases for example, pregnancy
Gastric volume reduction (Prokinetic drugs)	Metoclopramide	Ensure there is no intestinal obstruction
Anti-emetics	Metoclopramide, droperidol and granisetron Ondansetron	Given prophylactically if previous history of severe postoperative nausea and vomiting (PONV)
Anti-sialogogue	Atropine, glycopyrrolate	Not routinely given unless awake instrumentation
Depression of autonomic nervous system	Beta-blocker	May be indicated for patients with ischaemic heart disease
Prophylactic antibiotics: pregnancy, endocarditis, artificial joint replacement, pacemaker etc.	Penicillin, aminoglycosides and quinolones	Administered according to the protocol of institution
Analgesics	Morphine, pethidine, diclofenac, paracetamol	Indicated for severe pain, for example, fractures

*Source*: Please see the full reference list of the article Allman KG, Wilson LH. Pre-operative assessment and preparation for anaesthesia. Oxford textbook of anaesthesia. New York: Oxford University Press; 2011, for more information

## Record keeping and documentation

The documentation of the findings of preanaesthetic assessment just like other medical records is crucial for quality control and medico-legal reasons. However, the rate of documentation has been shown to vary depending on the criteria assessed, with airway assessments and peri-anaesthetic visit findings and incidents or accidents reportedly documented in less than 50% of patients.^32^ It is important to note that ‘if not documented, the assessment or intervention is assumed not done!’.

## Conclusion

The preanaesthetic assessment is an essential component of anaesthetic practice and allows for the systematic identification, categorisation and management of perioperative risks. The anaesthesiologists should discuss the findings with the patients and/or next of kin in an environment that is culturally appropriate and respectful, advise the patient on available anaesthetic choices and obtain informed consent. Considering the limitations of skills and equipment, primary care doctors in district hospitals should provide anaesthetic care within the scope of ASA-1 to 2. When there is an experienced Diplomate in anaesthesia, the scope may be extended to ASA 3 (E). In addition, all details of anaesthetic care should be documented in the medical records in accordance with professional and regulatory standards for optimal service outcomes.
